# Perspectives of Nonspecialists Delivering a Brief Depression Treatment in the United States: A Qualitative Investigation

**DOI:** 10.1186/s12888-023-04528-y

**Published:** 2023-01-13

**Authors:** Grace S. Woodard, Amanda Mraz, Brenna N. Renn

**Affiliations:** 1 Department of Psychology, University of Miami, Coral Gables, Florida USA; 2 Department of Psychology, University of Nevada, Las Vegas, 4505 South Maryland Parkway, MS 5030, Las Vegas, Nevada 89154-5030 USA

**Keywords:** Task sharing, Task shifting, Paraprofessional, Implementation outcomes, Qualitative methodology, Depression

## Abstract

**Background:**

Task sharing is an implementation strategy which increases access to services by training and supporting treatment delivery by nonspecialists. Such an approach has demonstrated effectiveness for depression and other mental health outcomes; however, few studies in high-income countries have examined nonspecialist providers’ (NSPs) perspectives of the acceptability, feasibility, and appropriateness of delivering mental health interventions. We examine qualitative reports of NSPs experiences delivering a brief structured behavioral intervention for depression (called “Do More, Feel Better” [DMFB]) to adults aged 55 and older.

**Methods:**

All NSPs (*N* = 4, 100%) who delivered DMFB participated in a focus group to probe their perceptions of the acceptability, feasibility, and appropriateness of both the intervention and their delivery experience as NSPs. Two coders analyzed the qualitative data from focus groups using codebook thematic analysis.

**Results:**

NSPs perceived the intervention and delivery experience to be acceptable, feasible, and appropriate. Qualitative results provided insight into specific barriers and facilitators which may be important to consider when planning to implement task sharing. Themes that emerged from qualitative data included supervision being highly acceptable and feasible, appropriateness of the intervention for NSPs, and the feasibility of tailoring the intervention to patient participants. NSPs also expressed difficulty managing emotional investment in patients’ success and providing therapy during a pandemic and racial violence in the US.

**Conclusions:**

Our results can inform future implementation and sustainment of task sharing interventions to expand access to care.

**Supplementary Information:**

The online version contains supplementary material available at 10.1186/s12888-023-04528-y.

## Introduction

In 2019, approximately one-fifth of American adults (20.6%) had a diagnosable mental health disorder [1]. Of those, less than half received any mental health services [1], meaning that 23 million adults were left untreated. Prevalence of common mental health conditions among older adults in the United States (US) mirrors that of the larger population with nearly one-third of this group living with symptoms of depression, yet relatively few receiving treatment [2]. Provider shortage is a leading cause of the mental health treatment gap in the US, with estimates that 7000 more providers are required to meet the need for services [3]. The unmet need for providers equipped to work with older adults is particularly stark [4].

A number of innovative methods to increase access to mental health treatment have been developed and applied to help close this treatment gap. These methods typically break away from the prevailing mental health model of receiving psychotherapy or pharmacotherapy from trained mental health professionals one-on-one in a clinical setting [5]. Task sharing, in which nonspecialist providers (NSPs) with no prior formal training as mental or physical health specialists are trained to effectively deliver treatment for mental or physical health conditions, is a promising implementation strategy which reduces the existing treatment gap by increasing the number of treatment providers [6–8]. The notion of expanding the healthcare workforce is not new; earlier writings on this issue outline the use of nonspecialists to perform essential medical tasks, often in the context of low- and middle-income countries (LMIC) [9] or in the case of nurses providing primary care health services as a substitute for physicians in high-income countries (HIC) [10]. The expanded use of such a workforce rose to prominence in addressing the HIV/AIDS crisis in sub-Saharan African and elsewhere [11] and has since been used successfully worldwide to deliver a variety of mental and physical health treatments across a range of populations, effectively reducing symptoms of depression, perinatal depression and anxiety, posttraumatic stress disorder, and alcohol use disorder [12–15].

Task sharing and task shifting models, including stepped care models and use of NSPs serving as primary providers, are more common in LMIC [13]. In the US, approximately 40% of NSPs served in auxiliary support roles due to barriers related to difficulty designing an appropriate role for NSPs, licensure and certification requirements, and a lack of necessary implementation supports for NSPs [13]. Existing NSP models in the US include community health workers, *promotores*, peer counselors and the like; while these roles expand access to care, such work typically focuses on psychoeducation, support, care coordination, and navigating resources [16]. However, attention to NSP-delivered behavioral health treatment in HIC is increasing [13]. One review [13] found that about half of the task sharing studies in the US focused on interventions delivered to children and families (51.3%), and most implemented novel community-driven interventions (69.2%) rather than evidence-based treatments (30.8%). Another review of nonspecialist-delivered interventions for perinatal mental health in HIC documented a growing evidence base, with 37.0% of studies delivering evidence-based psychological treatment [14]. Emerging literature has also tested NSP-delivered cognitive behavioral therapy (CBT) and related strategies for anxiety [17, 18] and depression [19–23] in older adults. This suggests NSP-delivered treatments are effective for a variety of populations. However, little is known about what types of implementation supports are most useful or most desired by NSPs while implementing such programs.

NSPs’ perspectives can directly inform researchers and policymakers by highlighting the implementation supports necessary to build and sustain this model of mental health treatment delivery. First, it is important to understand whether or not the NSPs find this model of delivery acceptable, feasible, and appropriate. Further, it is important to understand what aspects of the treatment, context, or implementation supports contributed to or detracted from these perceptions. However, few studies have examined NSPs’ perspectives of delivering mental health treatments. One such study in Kenya found that NSPs delivering an evidence-based trauma-focused treatment for youth found delivery highly acceptable, feasible, and appropriate [24]. A process evaluation of NSPs’ experience delivering a perinatal depression intervention in South Africa found wide variability in patient attrition and counselor fidelity to the intervention, which the authors contributed in part to varying levels of counselor self-efficacy and need for further training [25]. In the context of HIC, two qualitative studies in the US have investigated NSPs’ perceptions of delivering mental health services and found this approach was considered feasible [26] and appropriate [27]. However, the literature understanding the NSPs’ perspective of service delivery, particularly in the US context, is still extremely limited.

The current qualitative study evaluates NSPs’ (called “coaches”) perspectives delivering Do More, Feel Better (DMFB), a brief structured depression treatment based on behavioral activation, to depressed older adults (≥ 55 years old) via telehealth [23]. The US healthcare workforce is too small to meet the needs of a growing older adult population [4] and NSPs may be one way to bridge this gap. Older adults tend to prefer psychotherapy over pharmacotherapy [28–30] and evidence good outcomes with psychological therapies [31]; conversely, pharmacotherapy may inadequately treat older adults with apathy, persistent depressive disorder, or cooccurring cognitive impairment [32, 33]. Simplified evidence-based treatment approaches based on behavioral activation are well suited for NSP delivery [34] and acceptable to older adults [19]. Data supporting improved clinical outcomes is emerging, suggesting that DMFB delivered by nonspecialists significantly improved depressive symptoms and was acceptable to patient participants [21–23]. In the current study, coaches provided their perspectives of acceptability, feasibility, and appropriateness of delivery through a focus group. These three implementation outcomes were chosen because they are considered “leading indicators” of implementation success in formative research [35–37].

## Methods

### Intervention

DMFB is a brief behavioral treatment for mild-to-moderate symptoms of depression. Treatment is based on behavioral activation strategies, and coaches help patients to schedule activities and self-monitor their mood before and after engaging in activities to counteract the downward spiral of depression and activity avoidance [21]. This intervention was designed for and intended to be implemented by NSPs in a stepped care model, such as in primary care settings, where patients with complex presentations or who do not demonstrate treatment response can be more thoroughly evaluated and referred to higher steps of care (i.e., with a licensed provider). DMFB was originally designed to be delivered by volunteer NSPs in 12 weekly sessions [21], but was streamlined to six 30-minute sessions for this study [23] to be better suited to non-specialty settings such as primary care.

The program was rapidly shifted to telehealth delivery as the launch of the pilot study coincided with the onset of the COVID-19 pandemic. Specific adaptations included conducting all DMFB sessions over Zoom teleconferencing software or telephone (rather than in-person) and recording the sessions for later review. Patient participants were mailed packets with paper forms (i.e., symptom measures and session worksheets) to accompany each virtual session and provide tangible support for the intervention in the absence of in-person meetings. Coaches also received supervision regarding flexible application of behavioral activation strategies in the context of social distancing and restriction of usual activities.

### Procedures

#### Coach Recruitment and Training

Design, procedures, and primary outcomes of the parent study are detailed elsewhere [23]. In brief, coaches were invited to participate in the parent study after completing an advanced undergraduate university course offered through the departments of psychology and social work at the University of Washington in Seattle, WA. The nine-week course focused on basic clinical skills (e.g., empathic listening, structuring sessions) and trained students in delivering the DMFB protocol for depression. Training consisted of didactic methods, in-class instructor demonstrations, in-class role plays with real-time instructor feedback, and review of recorded role plays. A total of 18 undergraduates completed the course. Eight students were invited to participate in the parent trial based on demonstrated fidelity to the DMFB protocol assessed via recorded role plays. Four students enrolled as coaches. Coaches met with the supervising psychologist and principal investigator (B.N.R.) for a one-hour booster session prior to beginning intervention delivery for the parent trial.

#### Implementation, Supervision, and Fidelity Monitoring

Coaches (*N* = 4) delivered DMFB to three patients each (*N* = 12) between March and August 2020. Details about patient participants have been reported elsewhere [23]. In brief, they were on average 66.83 ± 10.39 years old; the majority were cisgender women (*n* = 11, 91.7%) and identified as either White (*n* = 8, 66.7%), Black/African American (*n* = 2, 16.7%), American Indian/Alaska Native (*n* = 1, 8.3%), and Multiracial (*n* =1, 8.3%). They all had elevated depressive symptoms as assessed by the Patient Health Questionnaire (PHQ-9 ≥ 10) [38]. Recordings of every first session and one randomly selected session were reviewed for fidelity. Weekly 30-minute individual supervision sessions were conducted via Zoom teleconferencing with B.N.R.

#### Current Study

After implementing DMFB with three patients in the parent study, NSPs were invited by the principal investigator to participate in a focus group for the current study. All four NSPs (100% of the sample) from the parent trial participated. The focus group was conducted over Zoom and led by the second author (A.M.), a cisgender woman graduate student researcher in clinical psychology, who was unaffiliated with the original study and not previously acquainted with the participants. Each participant was provided $50 as incentive for their participation in the focus group. All methods were carried out in accordance with relevant guidelines and regulations. All study activities were approved by the University of Washington institutional review board (IRB; protocol #00005877). Data for the current study were collected under approval for the parent trial, for which participants provided verbal informed consent, which was approved by the University of Washington IRB.

### Focus Group Methodology

Participants were asked seven questions in one 90-minute semi-structured focus group to understand aspects of the training, treatment protocol, and implementation that contributed to or detracted from the acceptability, feasibility, and appropriateness of DMFB. These constructs, as defined by Proctor and colleagues [36], imply judgement about the fit of an intervention, practice, service, technology, or other innovation. First, acceptability is the judgement that an intervention or practice is agreeable or satisfactory, with reference to either an individual provider or patient respondent (e.g., whether a provider perceives that the content of an intervention is credible). Feasibility is the perception of practicality—that is, whether an intervention or practice can be successfully used or carried out (e.g., a provider’s perception that a new intervention or innovation will be too timely or costly to implement). Appropriateness is the perceived relevance or compatibility of an intervention or practice with respect to a referent organization, setting, situation, patient presenting problem, or population (e.g., whether a provider perceives a new practice as meeting their patients’ needs). Coaches were asked questions in reference to the delivery experience broadly—that is, both the DMFB intervention specifically as well as the implementation strategy of using NSPs to deliver this treatment.

Participants were informed of the goals of the research. The interview guide was created by the first (G.S.W.) and third (B.N.R.) authors to directly query these three implementation targets and allow for emergent themes (see Additional File 1). The focus group was conducted in Fall 2020. It was audio recorded by the facilitator and sent to a third-party company for transcription (filler words removed; responses made anonymous) to allow for coding.

### Qualitative Analysis

The focus group transcripts were analyzed using thematic analysis [39]; specifically, using codebook thematic analysis [40, 41]. This offered a structured approach to coding (through use of a codebook) to facilitate the analysis of predetermined and emergent data. Our conceptual framework centered on the implementation outcomes described above regarding acceptability, feasibility, and appropriateness. Our analysis focused on “meaning-making” [41] (p. 591) from our interpretation of the experiences of coaches learning and delivering DMFB as NSPs as guided by these presuppositions about acceptability, feasibility, and appropriateness. The codebook was created through an iterative process. The first (G.S.W.) and second (A.M.) authors first independently read and re-read the transcripts to ensure familiarity with the data. They then independently conducted preliminary coding of the responses. Deductive coding focused on the predetermined conceptual framework of acceptability, feasibility, and appropriateness of NSPs’ delivery. The coding tree was arranged to prioritize parent codes of acceptability, feasibility, and appropriateness. Branches in the tree (subsequent codes) were examples of, contexts for, and causes of these parent codes. Coders also used open coding to allow for the detailing of emergent themes derived from the data. Coders met to compile and refine their codes into corresponding themes. Themes were reviewed by B.N.R. and verified via consensus discussion with all authors; transcripts were used as supporting evidence in any cases of discrepancies or disagreement. Transcripts were not returned to participants for comment. Illustrative quotes that exemplified the final themes were selected for inclusion in this manuscript to improve authenticity.

#### Reflexivity and Methodological Quality

We took several steps to strengthen the credibility and trustworthiness of the qualitative analysis [42]. The principal investigator (B.N.R.) had prolonged engagement with the coaches and had built trust and rapport, facilitating rich, detailed responses. However, to address reflexivity and to allow for more open and honest disclosure, the focus group facilitator (A.M.) was from a different institution and unacquainted with the coaches, and coaches were told the senior investigator would only have access to deidentified transcripts. All authors have experience with mental health; G.S.W. and A.M. are both doctoral students in clinical psychology and B.N.R. is a clinical psychologist. G.S.W. and B.N.R. are experienced with implementation science. B.N.R. has advanced training in qualitative analysis and experience leading such studies. All research procedures and analytic decisions were documented and discussed to maintain an audit trail. With regard to the issue of saturation, we sampled our entire population of coaches who were trained and delivered treatment (*N* = 4). Thus, further recruitment was not possible. To maximize code and meaning saturation [43], we employed a focus group to elicit all perspectives, and the facilitator used a mix of specific probes (to elicit content for deductive coding) and open-ended questions to fully understand the breadth and nuance of issues. The current study follows the COnsolidated criteria for REporting Qualitative research (COREQ) checklist [44].

## Results

### Participant Characteristics

Coaches all identified as cisgender women (*N* = 4, 100%), and on average were 25 years old (*SD* = 6.50). Two coaches identified as White (50%), one as Black (25%), and one as Multiracial (25%).

### Qualitative Results

#### Themes related to Acceptability

See Table 1 for a list of themes generated by the focus group. Theme 1: Acceptable nature of the behavioral rehearsal and immediate feedback during training, which facilitated understanding and satisfaction of delivering the intervention. Coaches reported that the focus on role play and receipt of instructor feedback during training was highly acceptable and helped with understanding the treatment. One coach said, *“I liked practicing with other students, and especially when [the instructors] gave us feedback right in the moment when we were role playing in the class. That was really helpful... It helped me learn really quickly.”*

**Table 1:** Themes generated from qualitative analysis of nonspecialists’ delivering a mental health treatment for depressed older adults

Implementation Outcome		Theme
Acceptability^a^		
*Perception of an intervention or practice as agreeable or satisfactory, with reference to either an individual provider or patient respondent*	Facilitators	Theme 1: Behavioral rehearsal and feedback during training
		Theme 2: Flexibility of intervention delivery
		Theme 3: Supervision
	Barriers	Theme 4: Structured nature of the intervention
		Theme 5: Lack of tools to support telehealth delivery
		Theme 6: Management of emotional investment with patients
		Theme 7: Desire for group supervision and emotional support
Feasibility^a^	Facilitators	Theme 8: Training and materials to support intervention delivery
*Whether an intervention or practice can be successfully used or carried out*	Barriers	Theme 9: Supervision as an implementation support
		Theme 10: Need for group supervision
		Theme 11: Time-limited sessions
		Theme 12: Desire for specialized training
		Theme 13: Desire for more challenges during training
Appropriateness^a^	Facilitators	Theme 14: Appropriate for coaches with and without a bachelor’s degree
*Judgment of relevance or compatibility of an intervention or practice with respect to a referent organization, setting, situation, or patient population*	Barriers	Theme 15: Intervention and delivery fit
		Theme 16: Need for more intensive specialty mental health services
		Theme 17: Mismatch between the brief structure of the intervention and perceived patient need
Emergent theme	Barrier	Theme 18: Stress and distress related to COVID-19 pandemic and anti-Black racism

Note: ^a^indicates term is defined based on Proctor et al., 2011 and Weiner et al., 2017

Theme 2: Appreciation for the flexible nature of delivering the intervention. Coaches reported that their experiences delivering DMFB with patients helped them to see the utility and flexibility of the treatment, which contributed to high perception of acceptability. For example,*“It was really funny starting out thinking like, ‘Oh, this [intervention] is way too simple. I don’t see how this would fit anybody with difficult things’ to actually being able to use it on somebody [who] was pretty complicated.”*

Theme 3: Supervision was highly acceptable. The main implementation support that contributed to acceptability was supervision. Coaches reported feeling safe and supported, and that when they needed to exercise flexibility within the treatment, it was supported by the supervisor, which made the treatment more acceptable to deliver.

*“My patient wasn’t just struggling with depression, she was also struggling with these really difficult [life] situations. I felt like I was going off script a little bit, and I was so nervous to meet with [the supervisor]. But then when [the supervisor] talked to me, she was like, ‘That was building the rapport with the patient, and it was showing empathy. None of that stuff was bad.' It fell into part of the protocol, even though it felt weird … And that just really reinforced the whole rest of the program for me.”*

There were aspects of DMFB and its implementation that did not seem as acceptable to coaches. Theme 4: Apprehension about the structured nature of the delivering the intervention. Some coaches had initial doubts prior to delivery, mainly due to the structured nature of the treatment. After training but prior to working with patient participants, some doubted the credibility or ability of a brief intervention to meaningfully effect change. However, this was remedied through applied experience delivering the intervention with patient participants (see Theme 3 above).

Theme 5: Lack of satisfaction with tools to support telehealth delivery. Two coaches noted that relying on paper worksheets and informational handouts were problematic, and because delivery of DMFB pivoted from in-person to telehealth during the COVID-19 pandemic, they wished the forms were available online.

Theme 6: Difficulty managing emotional investment in patient wellbeing. The main aspect which detracted from acceptability of treatment delivery was a strong emotional investment coaches made in their patients' success, which was difficult for them to manage in the context of a brief protocol. This was exacerbated by the COVID-19 pandemic and national reckoning with racial violence and anti-Black racism in Summer 2020.

*“I can definitely relate to not being prepared to deliver this during a pandemic. I think as much as we’re all feeling anxious and panicked, and whatever we’re feeling in the beginning of this, then I think a lot of heaviness was put on you in the session. And so all of my patients were really severely impacted by this pandemic, much more severely than I was, but it was really hard for me to shake that heaviness after the session, and it would just kind of be this extra weight on me that I think during maybe more normal times, when I was feeling more balanced, that would be easier [to manage].”*

*“I had a session the day that George Floyd was murdered, and that was really hard. That was really hard. I remember getting the news 30 minutes before my session, and I was just bawling. I was like, ‘Okay, do I cancel? What do I do?’ Managing my own emotions was really hard. ... [My patient, who was also Black,] had been tuning into the news, and she’s like, ‘All this [DMFB] stuff doesn’t feel relevant right now.’ And I was like, ‘Oh, I don’t know, but I’m also going through the same thing.’ I was like, ‘I’m also Black’ and bringing more of my identity into it really helped establish a connection … She grew up during segregation. She was telling me stories. It was a very valuable experience. And I had conversations with [my supervisor] like, ‘Okay. We have this connection, but I also don’t want to steer away from [DMFB] and her experience.’ I know that we can talk about these things and kind of just do that whole thing, but ... I don’t want to bring [in] and transfer any more trauma.”*

Theme 7: Desire for group supervision and more support managing emotional investment. Coaches reported feeling supported by talking through these common experiences in the focus group and expressed a desire for a space to talk with each other while delivering DMFB. One concrete suggestion was to create a group supervision space to learn from each other’s experiences delivering DMFB and managing (and normalizing) emotional distress.

*“This [focus group] is the first time that I’ve spoken to anybody else — like any of the other coaches — and heard about their experiences. And so I feel like it’s been so valuable to me, and I’m not even seeing any patients anymore. And so being able to add that throughout [the DMFB delivery] process would definitely be incredibly valuable, because this has been so interesting, and it’s been really neat to hear about other people’s experiences.”*

*“I totally agree [the focus group has been helpful]. And it’s just been nice to be like, ‘Oh, I’m not alone in that issue.’ Even just midway through to be like, ‘Wow, delivering therapy during a pandemic. This is intense. What do you guys do?’ I think that kind of stuff would have been really nice and just even if it’s not every week, just having a few seconds, because I think sometimes it did feel a little lonely.”*

#### Themes Related to Feasibility

Theme 8: Coaches felt well-prepared to begin delivery due to training experiences and provided materials. They reported that they were nervous to begin seeing patients, but once they did, they realized they were well prepared by training, and they were well supported by the forms and scripts which are a part of the DMFB treatment.

*“I think what was really comforting, I remember the first day, I was supposed to start, I was... really nervous, but then I remembered we had all of the worksheets. We had [session summary forms] and so it was just really concrete, which was something that I didn’t like at first. I was like, ‘This is sterile.’ It just feels very... formulaic, but when I was a little nervous, [the structure of the forms] actually really helped center me and ground me.”*

Theme 9: Supervision was the most commonly endorsed implementation support which made delivery of DMFB feasible for coaches. Specifically, supervision was safe and supportive for them to ask questions, and also helped them to tailor DMFB to each patient.

*“[My supervisor] was always just starting off with all the great things that I did [in session] that sometimes I didn’t recognize, and then thinking about ways to improve. So, it made it feel like a really safe space to be like, ‘I didn’t know what to do here. Help me.’ It really wasn’t evaluative.”*

*“And I also just want to say that I think one of the huge, huge reasons that we were able to be successful is because [our supervisor] is awesome.”*

There were aspects of DMFB and its implementation that did not seem as feasible to coaches. Theme 10: Many coaches endorsed a desire for group supervision to enhance their learning by getting exposure to different case presentations and to learn from each other’s cases.

*“I think since we were only seeing one patient at a time, or at least I did, that it was hard to imagine what other patient scenarios could come up. And so I think that maybe if we had heard scenarios that other people have dealt with and heard what they did and what [our supervisor’s] advice was, that it would have made it maybe easier. Like if that popped up for us in the future, we would have some sort of reference point rather than kind of starting from scratch.”*

Theme 11: Coaches reported feeling rushed in certain aspects of delivery due to the time-limited treatment. Coaches also reported desiring a built-in time for rapport building in the first session. They reported feeling rushed with the psychoeducation and goal setting in the brief time (30 minutes) allotted for the first session.

*“I really struggled with … allowing enough time to get in-depth enough where you can understand what’s happening and get to know your patient, and then going through the steps that had been laid out for us. I don’t think an hour would be necessary, but I think 30 to 45 minutes or allotting a little bit of extra time, maybe just even for the first session … And with some of my patients, [the time] was great– we were only 30 to 35 minutes. But then [for] others it was really challenging for me to have to interrupt when I felt that what we were talking about was valuable, and I wanted to hear it because it was overall going to help contribute to my ability to kind of tailor the activities and the goals and everything to the patient, but then I was always... very aware and cautious of going over that time limit.”*

Theme 12: Coaches desired more training on certain topics. Namely, they desired more training on the population they were working with (older adults), best practices of delivering mental health care via telehealth, and best practices for self-care as a clinician.

*“It was really challenging for me to be working with all of my patients that were so much older. And so there were times where someone would express something to me, and I just don’t have the life experience to relate to it … You can’t say, ‘I understand’ when they’re talking about something that is just so far off of anything I’ve ever experienced in my life. And so [my supervisor] would help me to kind of come up with different phrases and ways to express that you care.”*

*“Just the power dynamic [of patients] being so much older, and us being young, and them knowing that we’re not professionals, especially when it felt like we’re supposed to be being the professionals in a situation--that power dynamic was interesting.”*

*“I think that I started to learn a lot more about telemental health after I was done, and I was like, ‘Oh, yeah. That’s smart. We could have screen shared this or whatever.’ We weren’t fully set up to do that, so I don’t think it would have been super helpful if I had done that anyway. But just kind of thinking about best practices and telemental health would have been helpful, I think.”*

Theme 13: Coaches desired more challenging experiential learning as role plays with other classmates during training became routine. They suggested role plays with more challenging case vignettes, shadowing coaches delivering the treatment, or shortening training and delivering the intervention more quickly with close support.

*“With the [classroom] role play, something that I wasn’t really prepared for was how much emotional pain I could see [in] my patients...[like] when I listen to them speaking to me, or I saw them break down on the camera and then turn their camera off. They’re unable to kind of hold themselves together because that’s how much pain that they were in emotionally or physically. The role playing is great, and yes we can create scenarios, but … I think shadowing [other clinicians] is a great idea, because it’s so much more challenging to convey [in class role plays] the actual emotion that someone is going to be going through.”*

*“To me, I loved the role play in the beginning, and … the layers of role playing, but it definitely felt-- maybe week four or five-- that I wasn’t getting anything additional out of it. We were just trying to make up crazier stories and deal with more difficult patients, but they were mildly difficult compared to what we actually got.”*

#### Themes Related to Appropriateness

Theme 14: Coaches noted that delivery would be appropriate even for those without a bachelor’s degree. One coach said, *“I would say [with] a resounding 'yes' that definitely, people with BAs [bachelor of arts degrees]-- I would argue that people without BAs-- could deliver [DMFB].”*

Theme 15: The flexible nature of the treatment made it an appropriate fit for the needs of the patient participants and coaches. Coaches found DMFB appropriate to address myriad patient presentations and was able to be tailored to the patient’s individual goals and barriers. A coach said, *“Each patient was such a unique experience, and each individual I worked with was going through their own separate challenges. It required me to kind of tweak the style of [DMFB] that I was giving. I definitely had to make some judgment calls throughout the process.”*

There were some aspects of DMFB and its implementation which made it less appropriate for coaches. Theme 16: Coaches felt that patients needed referrals to more intensive services, but were not able to make them directly since they were not embedded within a healthcare setting. Coaches noted that some patients needed case management and/or more intensive treatment, perhaps because of chronic or comorbid mental health conditions. Research staff could provide community referrals for specialty mental health treatment or longer-term psychotherapy after the research protocol ended, but these referrals were difficult for patient participants to access and utilize due to their depression symptoms and barriers to accessing mental health treatment.

“*One of my patients had, in the middle of our treatment delivery, said that she had actually experienced a lot of trauma in her life. And so that really changed the way that [my supervisor] and I conceptualized this case. And so if we had someone else to refer to [directly], we would. I think two of my patients, some of our goals, we’re getting them into long-term care. And that was really hard for them, because they’re depressed, and they’re dealing with so much, and it’s during a pandemic, all sorts of extra challenges. I kept thinking, “Okay, if I were in the right setting, I could literally just [refer] them to a [trained] therapist, and they wouldn’t have to do anything. They just have to kind of show up.“ So, in some ways, I think that it would be easier to do it in that setting.”*

*“I think it might be easier for a bachelor’s level person to do this if they’re embedded in the right setting, because my patient was dealing with homelessness. Obviously, I’d be like, “Okay, this needs to go to a case manager, or someone more serious.””*

Theme 17: Coaches also noted the brief, structured manner of treatment presented challenges or a mismatch when a patient needed ongoing or more intensive psychotherapy or was bonded to the coach.

*“With my first patient, especially, I would have talked to her for two hours on Zoom. She just wanted someone really to talk to, I think. Saying goodbye to her after the six weeks is really hard, because I enjoyed our conversations. She was really struggling, and she admitted to me, I think, either during the second or final session, and she was like, ‘Well, I only feel comfortable talking to you and to my doctor about the things I’m going through.’ And so for someone to say that to me after only meeting with them once a week for six weeks, really kind of pained me. It was really hard to walk away from that.”*

#### Emergent Themes

Theme 18: Coaches additional sources of personal stress and distress during the pandemic and US reckoning with anti-Black racism made treatment delivery more challenging. Because this treatment was delivered in the beginning of the COVID-19 pandemic and during the US’s reckoning with anti-Black racism, coaches reported additional stressors from their own life impacting their delivery of DMFB. As covered above, coaches had difficulties coping with the emotional stress experienced by their patients due to the dual pandemic and racial violence. Specifically, a coach who identifies as Black reported worrying about experiencing racism from patients.

“One thing to add to touch on that is I was really afraid that my patients were going to bring things up and I was going to lose it [or not be] acting appropriately … And so I got really afraid when after [George Floyd] died and after everything was going on, and kind of, unfortunately, seeing the other side where people could be so f****** terrible about [his murder]. I wouldn’t be able to continue with a patient if they popped up with something that I thought was [racist]. I was going into that next week and [the] next couple sessions, I was so afraid of people talking [about racist sentiments].”

Other coaches reported challenges delivering DMFB due to the logistics of delivering the treatment from their home, patient boundary violations, additional caregiving responsibilities, and their own loss and stress from the pandemic.

*“I was doing this in my bedroom, and I lived in a house with eight other roommates. I was just in my bedroom because I couldn’t be downstairs or anything because there was constantly stuff going on. And one of my patients would point out things in my room, because I could only shut off my screen so much. And she would point out things in my room and kind of take away the focus from our session and put it onto me.”*

*“I didn’t start any of my patients until after the pandemic started. I’ve got two kids in the house, and I have an open concept [floor plan]. I was not prepared to be doing [DMFB] from home with two school kids during a pandemic.”*

## Discussion

Bachelor’s-level nonspecialist providers (NSPs), also called “coaches,” who implemented a low-intensity, brief behavioral treatment for depression found delivery to be acceptable, feasible, and appropriate. Moreover, these coaches rapidly pivoted to offering the program via telehealth during the early days of the COVID-19 pandemic, which speaks to the flexibility of service delivery but also made clear the need for unique supports related to telehealth delivery and emotional support. These preliminary results, combined with the patient outcomes reported elsewhere [23], suggest that a task sharing approach using trained bachelor’s-level nonspecialists as primary treatment providers is a promising approach to expand the US mental health workforce. To date, there are few projects using such a workforce to deliver mental health care in the US; there are even less data on the NSPs’ perspectives of this implementation strategy.

Our qualitative approach provided some context as to why coaches perceived DMFB favorably and suggest opportunities to further develop and refine training and delivery for such an approach. Training and implementation supports, including role play, feedback, and supervision, contributed to perceptions of acceptability and feasibility. Suggestions to further improve training and implementation supports include more training on the specific patient population (i.e., working with older adults) and delivery mode (i.e., best practices for telehealth), explicit attention to self-care as a clinician, and group supervision. Further research is needed to best design the delivery of such desired curriculum (e.g., classroom instruction versus training in field placements or apprenticeship-type models). The intervention (DMFB) and more generally the task sharing role still needs to be evaluated in the intended setting (i.e., primary care) to get more accurate data on implementation outcomes. Nonetheless, our coaches thought this NSP role and such a brief, structured treatment were a good fit for their training and professional goals.

Managing the emotional demands of clinical work—even in the context of a brief treatment—is an important consideration for sustaining this workforce. This concurs with the experience of other NSPs delivering a perinatal depression intervention in South Africa, who described how difficulties in their personal lives affected how they interacted with participants and how they dealt with their work [25]. Explicit attention to setting appropriate boundaries, practicing self-care, and methods for avoiding compassion fatigue and/or burnout in such NSPs are critical [45]. The suggestion for group supervision is an excellent consideration for implementation, as a means for both support and vicarious learning, as well as a cost-effective strategy with less demand than weekly individual supervision with an expert supervisor. One option is for a licensed mental health specialist to provide supervision to groups of NSPs. Another innovative option is to capitalize on the growing literature of peer supervision of NSPs. Evidence suggests that with time, training, and support of a structured therapy quality rating scale, peer supervisors can adequately rate NSP sessions [46], possibly as reliably as experts [47]. Such group and peer consultation models have been used successfully in task sharing trials [47–50], although it may be best when supplemented with expert support [47]**.** For example, an apprenticeship model for NSP-delivered mental health interventions in LMIC proposed a scaffolded approach to training and supervision. First, experts (often from outside contexts or countries) initially take responsibility for guiding and teaching the main skills to NSPs. Eventually though, this oversight transfers to trained NSPs who are local and are selected for an advanced role of supervisor to support intervention delivery and monitor quality assurance. These NSPs function as peer supervisors to other NSPs, with hopes of building and sustaining workforce capacity by leveraging individuals embedded in the local context [48]. Local peer supervisors may then continue to receive their own consultation or support from expert trainers on a less frequent basis, in order to provide scaffolded support [49]. Although reliance on outside experts may hinder scale-up, such models of group supervision and peer supervision may improve dissemination and sustainment [51] and are worthy of future research.

The DMFB treatment itself was rated favorably. Although coaches initially expressed some skepticism with the brief, structured treatment, their impressions changed when actually delivering the treatment with patient participants. Coaches suggested adapting session forms to make them available both digitally and in hardcopy, to allow for delivery in-person or via telehealth. Prior demonstrations of DMFB in this parent trial [23] and another delivered by trained volunteers in a senior center [21, 22] evidenced high patient acceptability, feasibility, and potentially meaningful improvements in clinical targets of depression, disability, and increased activity. This approach has the potential to expand access to effective and efficient mental health treatment–an important consideration when only 20% of individuals with depression in HIC receive minimally adequate care [52]. Additional research is needed to implement such a program, including examination of feasibility, appropriateness, and acceptability by setting. To scale up such work will also require more rigorous examination of implementation issues (e.g., reimbursement mechanisms, policy changes to credential such a role) from multiple community, regulatory, and clinical perspectives.

Themes highlighting coach stress and distress emerged from the data, related to the implementation of the parent study during spring and summer 2020 and ubiquitous sociopolitical and cultural influences of the COVID-19 pandemic and anti-Black racism in the US. These were both prominent influences in Seattle, WA (where the parent study was conducted) in 2020 and may have contributed to responses related to the expressed need for additional emotional support. The parent study was developed prior to the spread of the novel coronavirus and the murders of Ahmaud Arbery, Breonna Taylor, George Floyd, and other Black Americans in rapid succession in spring 2020. Rather than pause the study, investigators made the decision to shift the delivery to telehealth and offer services. First, the COVID-19 pandemic highlighted the dire need for available and accessible mental health services in the U.S. Depression symptom prevalence in the U.S. increased more than 3-fold during the COVID-19 pandemic in 2020 compared to 2017–2018 [53]; researchers undoubtedly attribute such psychological distress to the COVID-19 pandemic and related policies (e.g., social distancing) [54]. The parent study focused on older adults. While those aged 60 and older in the US reported lower levels of depression prevalence compared to younger aged adults during the pandemic, the proportion of older adults reporting significant depressive symptoms was still 13–15% and was more pronounced among those with poorer physical health and lower socioeconomic status [53, 55]. Clearly, many preexisting inequalities in psychological distress and access to care remained and were further complicated by the pandemic. In a very pragmatic way, social distancing policies made clear the need for appropriate telemental health supports (both for this parent study and in general). It also required flexibility on behalf of the coaches, ranging from providing telemental health services from home to helping patient participants brainstorm and schedule activities when many usual activities and routines were severely disrupted. Finally, the coaches themselves were dealing with similar psychosocial stressors as the patient participants. Uncertainty reigned, some had to relocate (e.g., communal and university housing abruptly closed) or faced isolation and loss of social supports, and some were worried about family members (and their own) susceptibility to infection. Add to this the renewed visibility of the Black Lives Matter movement and related social justice and anti-racism discourse, both locally and nationally. Racism has long been an ignored topic in psychotherapy, and discussion and validation of such experiences — especially anti-Black racism—is highly variable and largely based on therapists’ own multicultural competence, cultural humility, and internal response [56]. Our findings from this preliminary examination of NSP experiences delivering an intervention make clear the need to train NSPs and clinicians in anti-racist, anti-oppressive, structurally responsive, and culturally humble practices [57]. This applies specifically to the experiences of those from Black and African American communities, as well as to Indigenous, multiracial, and other people of color. Importantly, the results of our interviews also highlight a lesser discussed issue of supporting Black clinicians [58].

### Limitations

Methodological considerations include a small sample size of four coaches and only one supervisor (B.N.R.), who also conducted the training and served as principal investigator. Virtually no work has been done on the experience of NSPs in US settings, despite growing attention to this model to expand access to mental health care. No true standards regarding sample sizes in qualitative research exist [59, 60]; thus, determining sample size is to some degree a pragmatic exercise of what an expected or minimum sample size will be. This can be guided in part by the aims of a study. Although all NSPs who participated in the parent trial were interviewed, their perspectives may not necessarily mirror those of a larger group. Moreover, the coaches who participated in the parent trial and the current study represent a small subset of all the students who enrolled in the training program in the parent study [23]. For example, these were students who were interested in applying to graduate school in mental healthcare professions and had displayed a high degree of academic and interpersonal aptitude. As NSP roles begins to take shape in the US and other HIC contexts, it is crucial to understand how learner characteristics may be best assessed or supported to ensure success and to facilitate diversity in the workforce. Other limitations include a lack of other informants in the qualitative assessment. Although the parent study [23] assessed a few metrics of patient acceptability, we did not include a thorough assessment of patient or other relevant partner (e.g., policymaker) experiences. Despite these limitations, we believe such an exploratory investigation of coach experiences is an important contribution based on the relatively few studies in HIC which examine the perspectives of NSPs. Future task sharing demonstrations should include process evaluations—including evaluating the NSPs’ experience—to inform implementation effects and facilitate interpretation of outcomes.

## Conclusion

This study is unique for several reasons. First, it is one of few studies in the US which used trained bachelor-level NSPs as the primary providers of treatment. Second, to our knowledge, is the first study exploring NSPs’ perceptions delivering evidence-informed treatment for depression in the US. Lastly, it is one of few studies to investigate NSPs’ perspectives on delivering a mental health treatment via telehealth during the COVID-19 pandemic. As seen in other global mental health contexts, task sharing for common mental health conditions is a potential option to increase access to services and maximize resources in the US. In this preliminary study in Washington State, bachelor’s-level NSPs perceived acceptability, feasibility, and appropriateness of a brief, structured behavioral treatment delivered to individuals with depressive symptoms. Future work holds promise for leveraging such a NSPs workforce to scale up mental health services.

## Supplementary Information

**Additional file 1.**

## Data Availability

The datasets analyzed during the current study are available from the corresponding author on reasonable request.

## Abbreviations

CBT: Cognitive Behavioral Therapy
COVID-19: Coronavirus disease of 2019
DMFB: “Do More, Feel Better”
HIC: High-income countries
IRB: Institutional review board
LMIC: Low- and middle-income countries
NSP: Nonspecialist provider
US: United States of America
