# Supplementary material for: Perspectives of Nonspecialists Delivering a Brief Depression Treatment in the United States: A Qualitative Investigation
Source: BMC Psychiatry. 2023 Jan 13;23:32. doi: 10.1186/s12888-023-04528-y (PMC9839228; doi:10.1186/s12888-023-04528-y)
Supplement: Supplementary file 1 — Additional file 1. [file 12888_2023_4528_MOESM1_ESM.docx]

**Interview Guide for “Do More, Feel Better” Focus Group:**

1. Let’s start with your general impressions and experiences with Do More, Feel Better. Think of the entire time you have been involved in learning and delivering Do More, Feel Better, from when you first heard about it to delivering your last session. Tell me about this experience.
2. What helped you deliver Do More, Feel Better? These could be things done by you, people who assist the program, or your supervisor.
   1. Probe: What about supervision was helpful in your delivery of Do More, Feel Better?
3. What were the challenges that you experienced while delivering Do More, Feel Better?
   1. For each one, was this challenge overcome?
   2. If yes, how?
4. What training challenges impacted your delivery of Do More, Feel Better?
   1. What was done to overcome these challenges related to training?
5. What were the challenges related to supervision that impacted your delivery of Do More, Feel Better?
   1. What was done to overcome these challenges related to supervision?
6. What is your opinion of Bachelor’s-level individuals providing Do More, Feel Better in the future?
7. Is there anything else you can think of that could be done to improve the implementation of Do More, Feel Better?
   1. Probe: any modifications to the protocol, delivery format, worksheets, etc.
